# Integrated Cognitive Processing Therapy and Relapse Prevention for Co-Occurring PTSD and Alcohol Use Disorder: A Case Series Examining Acceptability and Initial Efficacy

**DOI:** 10.3390/bs15081000

**Published:** 2025-07-22

**Authors:** Anka A. Vujanovic, Amber M. Jarnecke, Fiorela Ruiz, Kayla E. Hall, Katharine Roberts, Tanya C. Saraiya, Sudie E. Back

**Affiliations:** 1 Department of Psychological and Brain Sciences, Texas A&M University, College Station, TX 77840, USA; fiorelaruiz@tamu.edu (F.R.); kaylahall@tamu.edu (K.E.H.); 2 Department of Psychiatry and Behavioral Sciences, Medical University of South Carolina, Charleston, SC 29403, USA; jarnecka@musc.edu (A.M.J.); roberka1@musc.edu (K.R.); saraiya@musc.edu (T.C.S.)

**Keywords:** trauma, PTSD, alcohol, treatment, integrated, cognitive processing therapy, relapse prevention, comorbidity

## Abstract

Posttraumatic stress disorder (PTSD) and alcohol use disorder (AUD) often co-occur and present significant treatment challenges. Cognitive Processing Therapy (CPT) is a widely used, efficacious treatment for PTSD, but the application of CPT among individuals with co-occurring PTSD/AUD has been limited. To address this gap, we developed a novel, 12-session trauma-focused treatment that combines CPT with Relapse Prevention (RP) for AUD (CPT+RP). This paper describes CPT+RP and presents preliminary outcomes from the first six participants enrolled in a larger, ongoing multisite clinical trial of CPT+RP. PTSD symptoms were assessed using the Clinician-Administered PTSD Scale for DSM-5 (CAPS-5) and PTSD Checklist for DSM-5 (PCL-5). The Timeline Follow-Back (TLFB) assessed frequency (percent days drinking; PDD) and quantity (drinks per drinking day; DDD) of alcohol use, and craving was measured using the Penn Alcohol Craving Scale (PACS). The Client Satisfaction Questionnaire measured acceptability. Pre- to post-treatment reductions were observed in PTSD symptoms (ΔMCAPS-5 = 14.00; ΔMPCL-5 = 20.50), frequency and quantity of alcohol use (ΔMPDD = 38.65; ΔMDDD = 6.24), and craving (ΔPACS = 6.17). Most participants achieved clinically significant improvement in their PTSD symptoms and acceptability was high. Although preliminary, the findings suggest the new CPT+RP intervention is feasible, acceptable, and a promising treatment innovation for co-occurring PTSD and AUD.

## 1. Introduction

Alcohol is the most frequently used and misused substance in the United States (U.S.) (Grant et al., 2017; Grant et al., 2015). AUD frequently co-occurs with posttraumatic stress disorder (PTSD) (Back et al., 2024; Walter et al., 2018), a comorbidity that is complex and marked by a more costly, severe, and chronic clinical course, when compared to either disorder alone (Vujanovic & Back, 2019) of AUD and PTSD has been well-documented, with studies showing that 30% to 63% of individuals with AUD also have PTSD (Jacobsen et al., 2001; Ouimette et al., 2005; Read et al., 2004; Seal et al., 2011; Stewart et al., 2000). The onset of PTSD tends to precede, and thus may increase risk for, the development of AUD (Bountress et al., 2021). Individuals with PTSD/AUD often report using alcohol as a coping strategy, in general, and specifically to mitigate distressing PTSD symptoms (Back et al., 2024). If left untreated, individuals with PTSD/AUD are at increased risk for developing additional psychiatric conditions (e.g., depression and suicidality), medical problems, and social and occupational impairment (Norman et al., 2018; Simpson et al., 2019). Moreover, untreated PTSD is a risk factor for excessive alcohol use and relapse (Norman et al., 2018). Both PTSD and AUD, therefore, in an integrated fashion may help patients achieve long-term positive outcomes.

Treatment utilization is low among individuals with PTSD/AUD and less than 20% receive care for both AUD and PTSD (Simpson et al., 2019). Meta-analytic studies and systematic reviews indicate that trauma-focused therapies (i.e., treatments for which the central focus is trauma (VA/DoD Clinical Practice Guideline Work Group, 2023)), such as Cognitive Processing Therapy (CPT), are more efficacious than non-trauma-focused interventions among individuals with co-occurring PTSD and substance use disorders (Back et al., 2024; VA/DoD Clinical Practice Guideline Work Group, 2023; Hien et al., 2024; Roberts et al., 2015; Simpson et al., 2017). While integrated non-trauma-focused interventions, such as Seeking Safety, are widely used, clinical trials demonstrate that non-trauma-focused treatments are no more effective than treatment-as-usual or substance use only treatments (VA/DoD Clinical Practice Guideline Work Group, 2023).

At present, only one integrated, trauma-focused intervention for co-occurring PTSD and substance use disorders is available. Concurrent Treatment of PTSD and Substance Use Disorders Using Prolonged Exposure (COPE) (Back et al., 2014) combines Prolonged Exposure (PE), which includes imaginal and in vivo exposures (i.e., repeatedly recounting the trauma memory and approaching trauma-related cues in the environment, respectively) to treat PTSD alongside cognitive behavioral therapy (CBT) for substance use disorders. Data from numerous randomized clinical trials demonstrate the safety, feasibility, and efficacy of COPE in reducing PTSD and substance use (Back et al., 2019; Mills et al., 2012; Persson et al., 2017). In addition, a clinical trial by Norman and colleagues (Norman et al., 2019) comparing COPE to Seeking Safety found that COPE led to significantly greater reductions in PTSD severity, higher rates of PTSD diagnostic remission (51.5% vs. 17.2%), and greater improvements in trauma-related guilt and residual PTSD symptoms (e.g., persistent avoidance, hypervigilance, sleep and concentration difficulties) (Tripp et al., 2020). Rates of AUD remissions were also higher in COPE than Seeking Safety (45.5% vs. 37.5%) (Norman et al., 2019). Despite these important advances in trauma-focused integrated treatments for PTSD and substance use disorders, substantial room exists to enhance treatment options for PTSD/AUD. There is a need to expand the portfolio of evidence-based, trauma-focused integrated treatments to enhance patient and provider choice and further improve treatment outcomes and retention.

Clinical trials targeting the treatment of PTSD alone demonstrate that both CPT and PE are effective (Gutner et al., 2013; Powers et al., 2010; Resick et al., 2002; Rutt et al., 2018) and yield comparable durability at 10 years post-treatment (Resick et al., 2012; Wachen et al., 2014). Most recently, Schnurr and colleagues conducted a multisite randomized clinical trial comparing CPT and PE among military veterans (N = 916; 461 randomized to CPT and 455 to PE) across 17 different Veterans Affairs (VA) medical centers (Schnurr et al., 2022). Both trauma-focused treatments led to significant and meaningful reductions in PTSD symptoms. While reductions in PTSD symptoms were statistically greater in PE than CPT, the differences were not clinically significant. Client satisfaction and the safety profile of the treatments were also similar across PE and CPT. The authors highlight the importance of shared decision-making with patients given that both interventions resulted in significant and meaningful improvements. Notably, although the sample was not a comorbid PTSD and substance use disorder sample, heavy drinking and drug use were measured using the Brief Addiction Monitor (BAM); neither treatment led to significant pre-post reductions in alcohol use. The lack of change observed in substance use when using PTSD-only focused treatments suggests that it may be necessary to integrate and directly address co-occurring substance use to achieve significant improvements in both outcomes.

Other studies examining CPT among patients with PTSD have shown it leads to a reduction in suicidal ideation, hopelessness, and guilt-related cognitions (Gallagher & Resick, 2012; Resick et al., 2008; Schumm et al., 2015). CPT can also be applied in both individual and group formats, although the individual therapy format shows greater efficacy (Resick et al., 2017). Because CPT does not involve revisiting the memory of the traumatic event, as is done in PE, it is less reliant upon patients’ memories, which can be affected by numerous factors (e.g., age of the trauma, loss of consciousness or injury during the trauma). These features not only make CPT an accessible treatment option for a wide range of individuals with PTSD but also underscore the importance of developing an integrated, trauma-focused treatment that utilizes CPT for the PTSD treatment component.

Moreover, research on provider preferences demonstrates enthusiasm for a manualized CPT+RP intervention (Vujanovic et al., 2023). In the absence of such an intervention, many clinicians resort to piecing together available treatment protocols on their own to treat their patients with PTSD/AUD (Vujanovic et al., 2023). This approach has numerous shortcomings and may result in ineffective and variable treatment implementation and outcomes. Indeed, the lack of a manualized intervention that incorporates CPT for the treatment of co-occurring PTSD and AUD has been a major barrier in the field, limiting research on trauma-focused integrated interventions and limiting options for providers and patients (Vujanovic & Back, 2019; Vujanovic et al., 2018).

To address this need, Vujanovic and colleagues developed a highly novel and scalable trauma-focused treatment that represents the integration of two evidence-based treatments for PTSD and substance use disorders: CPT and RP (Asmundson et al., 2019; Marlatt & Donovan, 2005; Vujanovic et al., 2018). The new trauma-focused CPT+RP intervention is a streamlined treatment with strong potential for dissemination in diverse clinical settings and delivered by a range of clinical providers. The first large-scale RCT to address co-occurring PTSD/AUD using the new CPT+RP intervention, as compared to RP alone, is currently underway (Vujanovic & Back, 2024). This project will also help bridge the gap between currently available treatment options and provider preferences (Simiola et al., 2019). Moreover, the project incorporates ecological momentary assessment (EMA) which will provide novel insights regarding symptom interplay between PTSD symptoms, alcohol use and craving, and serve as a fertile basis for theory-driven modeling in future research.

The present case series presents data on the first six participants to consecutively complete the new CPT+RP treatment. We hypothesized that individuals randomized to CPT+RP, as compared to RP, would experience greater reductions in PTSD symptoms, alcohol use (frequency and quantity) and depression. We also hypothesized that individuals receiving the CPT+RP treatment would report high levels of treatment acceptability.

## 2. Materials and Methods

### 2.1. Participants

Participants were enrolled in a larger, ongoing multi-site clinical trial (NCT05959434) comparing the integrated CPT+RP treatment to RP alone (Vujanovic & Back, 2024). Study materials and methods were approved by the Texas A&M University and Medical University of South Carolina Institutional Review Board (IRB). All participants signed an IRB-approved consent form before any study procedures occurred. Inclusion criteria includes (1) aged 18 years or older; (2) able to provide informed consent in English; (3) meet DSM-5 criteria for PTSD based on the Clinician Administered PTSD Scale for DSM-5 (CAPS-5; (Weathers et al., 2018)); and (4) meet DSM-5 criteria for moderate to severe AUD based on the Quick Structured Clinical Interview for DSM-5 Disorders (QuickSCID-5 (First & Williams, 2021)). In addition, participants must report 3 or more heavy drinking days (HDD), defined as >4 drinks in one day for females and >5 drinks per day for males, in the past month, or consume > 14 drinks per week for females or >21 drinks per week for males for at least 2 weeks in the past month. Other substance use is permitted, but alcohol must be the primary substance of concern. Individuals taking psychotropic medications are required to be maintained on a stable dose for at least four weeks prior to study start. Exclusion criteria include (1) significant alcohol withdrawal symptoms as evidenced by a score greater than 8 on the Clinical Institute Withdrawal Assessment for Alcohol Scale–Revised (CIWA-Ar) (Sullivan et al., 1989); (2) current or lifetime psychotic or bipolar disorder; (3) imminent risk of suicidal or homicidal behavior; (4) pregnant or breastfeeding; and (5) currently enrolled in an evidence-based treatment for PTSD or AUD. Participants included in this case series including the first 6 participants who completed all 12 sessions of CPT+RP and the 3-month follow-up assessment at the time of data analysis (January 2025).

### 2.2. Procedures

Participants were recruited nationally using online advertisements as well as locally through clinical referrals. Following a brief screening, potentially eligible participants were scheduled for a more comprehensive baseline appointment. Eligible participants were then randomly assigned to complete 12 sessions (90 min each) of either CPT+RP or RP with twice weekly individual sessions over six weeks. Ineligible participants were referred clinically for treatment. Only the CPT+RP participants are included in this preliminary case series. Table 1 includes the CPT+RP session content for each of the 12 sessions.

Study therapists in the trial are required to have a master’s or doctoral degree, complete a two-day training, and participate in weekly ongoing supervision. Study therapists deliver only one of the interventions (CPT+RP or RP) in the project. Participants are given the option to participate via telehealth (virtually) or in person, and all participants included in this case series elected to complete the study via telehealth.

### 2.3. Measures

#### 2.3.1. Demographic Questionnaire

Participants were asked to self-report demographic information (e.g., age, race and ethnicity, sex and gender, relationship status, education) using a form created for this study.

#### 2.3.2. Clinician Administered PTSD Scale for DSM-5 (CAPS-5; (Weathers et al., 2018))

The CAPS-5, a 30-item structured interview, was used to diagnose PTSD and evaluate PTSD symptom severity. The CAPS-5 assesses the presence of each PTSD symptom using a 5-point Likert scale (0 = *Absent* to 4 = *Extreme/Incapacitating*), as well as the onset of symptoms and clinically significant distress and impairment due to symptoms. Total scores range from 0 to 80 with higher scores indicating more severe symptomatology. The CAPS-5 past-month version was administered at baseline, session 12, and the follow-up visit.

#### 2.3.3. Life Events Checklist for DSM-5 (LEC-5; Weathers et al., 2013a)

The LEC-5, a self-report questionnaire, was used at baseline to assess lifetime traumatic experiences and identify an index traumatic event. The LEC-5 asks about 16 potentially traumatic events (e.g., natural disaster, combat, sexual assault, transportation accident) as well as an additional item assessing for ‘other’ potentially traumatic events not listed. For each event, respondents indicate if the event happened to them, they witnessed it, they learned about it happening to a close friend or family member, they experienced it as part of their job, they are not sure, or if it does not apply to them.

#### 2.3.4. PTSD Checklist for DSM-5 (PCL-5; Weathers et al., 2013b)

The PCL-5 is a 20-item self-report questionnaire that measures PTSD symptom severity. Keeping their index trauma (identified by the LEC-5) in mind, participants rate each item on a 5-point Likert scale (0 = *Not at all* to 4 = *Extremely*) in regard to how often they have been bothered by the symptom. Total scores range from 0 to 80, with higher scores indicating greater PTSD symptom severity. A score of 31 or higher indicates a probable PTSD diagnosis. The PCL-5 was administered at baseline and at each study visit to monitor PTSD severity.

#### 2.3.5. Timeline Follow-Back (TLFB; Sobell & Sobell, 1992)

The TLFB is a calendar-based assessment that collects information regarding substance use over a specific timeframe and provides information on the amount, frequency and pattern of use. For the current study, daily alcohol consumption was measured for the past 60 days at baseline, weekly during treatment, and during the follow-up period.

#### 2.3.6. Penn Alcohol Craving Scale (PACS; Flannery et al., 1999)

The PACS is a 5-item self-report measure that assesses frequency, intensity, and duration of cravings for alcohol. The total score ranges from 0 to 30 and a PACS score ≥15 indicates clinically significant alcohol craving (Hartwell et al., 2019).

#### 2.3.7. Patient Health Questionnaire-9 (PHQ-9; Kroenke et al., 2001)

The PHQ-9 is a 9-item, self-report measure used to screen and monitor the severity of depressive symptoms. Respondents rate each item using 4-point Likert scale (0 = *Not at all* to 3 = *Nearly every day*). Items query major symptoms of depression such as anhedonia, feeling down, sleep disturbances, fatigue, appetite changes, and suicidal thoughts. Total scores range from 0 to 27, with scores of 5–9 indicating minimal symptoms, 10–14 mild depression, 15–19 moderately severe depression, and 20 or higher severe depression. The PHQ-9 was administered at baseline and each study visit.

#### 2.3.8. Client Satisfaction Questionnaire (CSQ; Larsen et al., 1979)

The CSQ, an 8-item self-report measure, assessed participants’ general level of satisfaction and perceived quality of the therapeutic services they received. Each item is rated using a four-point Likert scale from 1 (low satisfaction) to 4 (high satisfaction). Items are summed for a total score (range from 8 to 32), with higher scores indicating greater satisfaction.

### 2.4. Data Analysis

Total scores on measures of PTSD (CAPS-5, PCL-5), craving (PACS), and depression (PHQ-9) were calculated for each assessment timepoint at which the variable was collected. For the TLFB, summary variables reflecting frequency of alcohol consumption (percent days drinking; PDD) and quantity of alcohol consumed (drinks per drinking day; DDD) were created and used in the analyses. The percent of heavy drinking days (PHDD), defined as ≥4 drinks in one day for females and ≥5 drinks per day for males, was also examined. Difference score calculations were used to examine change in symptoms or alcohol use from pre- (baseline assessment) to post-treatment (session 12). When available, difference scores were compared with previously established criteria for reliable change. Changes in symptoms and alcohol use over time (e.g., baseline, treatment phase, follow up phase) were also examined graphically to ascertain overall trends in the data. The average CSQ score was examined to evaluate the acceptability of the CPT+RP treatment among participants.

## 3. Results

### 3.1. Demographics and Baseline Clinical Characteristics

The average age of participants was 42.67 years old (*SD* = 16.96) and 83.3% (5/6) identified as women. Five participants identified as White, one as Black, and one as Hispanic/Latinx. Participants resided in the Southwest, Southeast, Northeast, and Midwest geographical regions of the U.S. Three participants were single/never married, one was married, and two were divorced. No participants were employed full-time; one participant was employed part-time, two were unemployed, two were students, and one was retired.

The average CAPS-5 and PCL-5 total scores at baseline were 33.33 and 46.17, respectively. Participants endorsed experiencing an average of 6.67 different types of traumatic events. Index traumas were physical assault (*n* = 3), adult sexual assault (*n* = 2), and childhood sexual abuse (*n* = 1). All participants met DSM-5 criteria for current AUD with the majority (5/6) meeting criteria for severe AUD and one for moderate AUD. Participants reported consuming alcohol on half of the days (50.56%) in the past 60 days, with an average of 7.29 drinks per drinking day. The average PACS total score of 17.33 at baseline indicates clinically significant craving and the PHQ-9 score of 15.17 indicates moderately severe depression.

### 3.2. Clinical Outcomes

Table 2 provides average symptom scores for PTSD severity, alcohol use frequency and quantity (PDD, ADD, and HDD), alcohol craving, and depressive symptoms at baseline, mid-treatment (except for the CAPS-5 which was not assessed at mid-treatment), post-treatment, and 3-month follow up.

#### 3.2.1. PTSD Severity

**Figure 1:**
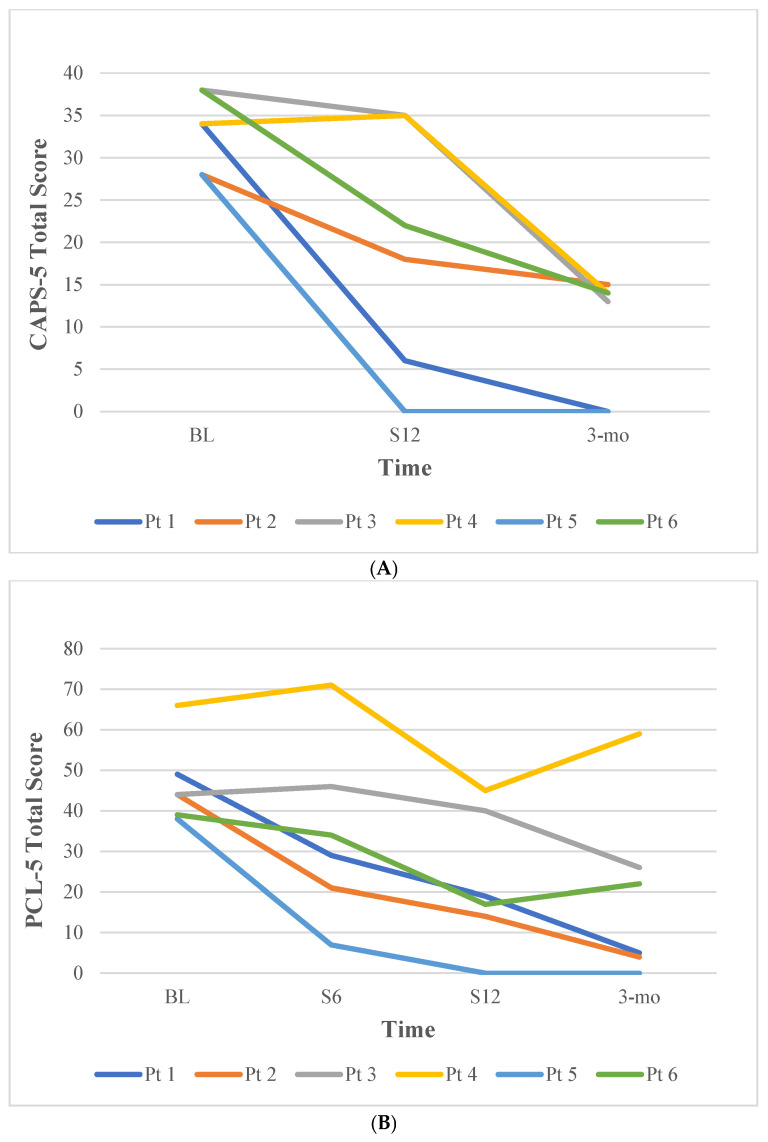
Change Over Time in PTSD Symptoms for Individual Participants. Note. (**A**) Change in CAPS-5 total scores. (**B**) Change in PCL-5 total scores. Pt = participant. BL = baseline; S6 = session 6; S12 = session 12; 3-mo = 3-month follow-up.

Examination of pre-to-post treatment differences in PTSD severity from baseline to session 12 revealed reductions over time. On average, participants had a 14.0-point reduction (*SD* = 12.31) on the CAPS-5 total score from 33.33 at baseline to 19.33 at session 12, below the cutoff for probable PTSD diagnosis. At 3-month follow up, CAPS-5 scores were further reduced (M = 17.00). For the PCL-5, a 20.5-point reduction (*SD* = 9.75) was observed, reducing from a score of 46.67 at baseline to 22.50 at session 12. Similarly, scores on the PCL-5 continued to lower at the 3-month follow-up visit (M = 19.33). Based on established thresholds, three of six participants met criteria for reliable change in PTSD symptoms based on the CAPS-5 (i.e., ≥13-point reduction; (Marx et al., 2022)), and four of six participants met criteria for reliable change in PTSD symptoms on the PCL-5 (i.e., ≥18-point reduction; (Gyani et al., 2013)) at session 12. As shown in Figure 1, treatment gains in PTSD reductions were maintained for most participants during the follow-up period, and PTSD severity at follow-up was lower for all participants compared to baseline.

#### 3.2.2. Alcohol Use and Craving

Examination of pre-to-post treatment differences in percent days drinking (PDD) any alcohol, percent heavy drinking days (PHDD), and the number of drinks consumed per drinking day (DDD) revealed reductions over time. On average, participants experienced a 38.65%-point reduction in PDD (*SD* = 35.07) from drinking 50.56% of the time at baseline to 11.91% of the time at the end of treatment. In addition, the frequency of heavy drinking reduced from 48.33% of days at baseline to 11.90% of days at the end of treatment, representing a 36.43%-point reduction in PHDD (*SD* = 35.26). Regarding quantity of alcohol, DDD decreased by 6.24 drinks (*SD* = 2.47), reducing from 7.29 drinks per day at baseline to 1.05 drinks per day at the end of treatment. Five out of six participants were completely abstinent from drinking by the end of treatment. Average scores on PDD, PHDD, and DDD were lower at the 3-month follow-up compared to baseline (see Table 2).

The PACS, which measures craving for alcohol, reduced 6.17 points (*SD* = 11.79) over time, from 17.33 at baseline to 11.17 at the end of treatment, falling below the cutoff for clinically significant craving. At the 3-month follow-up, the PACS was further reduced to 7.50 (see Table 2).

#### 3.2.3. Depressive Symptoms

On average, participants experienced a pre-to-post treatment reduction of 5.00 points (*SD* = 4.47) on the PHQ-9, from 15.17 at baseline to 10.17 at the end of treatment, representing a change from moderately severe depression to mild depression. Half of the participants (3/6) also met criteria for reliable change in depressive symptoms (≥5.20-point reduction; (Bryan et al., 2016)) by session 12. As shown in Table 2, scores on the PHQ-9 continued to lower at 3-month follow-up (M = 7.50).

## 4. Discussion

This case series provides preliminary evidence supporting the feasibility, acceptability, and potential clinical utility of a new integrated, trauma-focused CPT+RP intervention. By combining elements of CPT and RP together in each session, this treatment addresses symptoms of both disorders simultaneously. This allows for greater efficiency and greater conceptual cohesion of the dual diagnosis, enhancing patient’s understanding of how one disorder affects or exacerbates the other, and promoting improvement in both conditions using on treatment. To date, only one other trauma-focused integrated intervention has been developed, making the new CPT+RP treatment an important contribution to the literature and a promising advancement for clinical practice.

Across the first six participants to complete the CPT+RP intervention in the context of an ongoing randomized clinical trial, the results demonstrate that CPT+RP led to meaningful reductions in both clinician-administered and self-reported PTSD symptoms. Severity scores decreased, on average, by 14 points on the CAPS-5 and 21 points on the PCL-5, with most individuals achieving reliable and clinically significant change. Further, the improvements seen during treatment were sustained or further improved at the 3-month follow-up. Notably, end-of-treatment CAPS-5 scores fell below the threshold for probable PTSD for the majority of participants. The magnitude and durability of these reductions in PTSD severity are consistent with prior findings of CPT (Schnurr et al., 2022) and extend this evidence base to patients with co-occurring AUD.

Alcohol-related outcomes also demonstrated meaningful reductions over time. Participants reduced the frequency of drinking from 50% of the time to only 11% percent of the time, and the number of drinks consumed on the days that participants drank reduced by over six drinks per day, falling to approximately one drink per drinking day. In addition, most participants achieved abstinence from alcohol by the end of treatment. This is important to note, especially given that participants are not required to have a treatment goal of abstinence to enroll. Reductions in frequency and amount of alcohol use were accompanied by decreases in alcohol craving (PACS) and heavy drinking days (TLFB), with continued reductions observed in both over the follow-up period.

The addition of Relapse Prevention (RP) strategies to a trauma-focused therapy like CPT represents an important advance in integrated treatment approaches. By directly targeting mechanisms that may contribute to the development or maintenance of both PTSD and alcohol misuse (e.g., avoidance, negative cognitions, maladaptive coping), CPT+RP may effectively address both PTSD and AUD. Different from PE-based approaches for PTSD, CPT does not include imaginal or in vivo exposures, making it an accessible option for individuals who do not remember the trauma, those who are unwilling to engage in exposures, and/or those for whom a PE-based approach was not optimally effective. Moreover, many providers are trained in CPT but not PE, making it important to have an evidence-based integrated CPT protocol for individuals with PTSD and comorbid AUD.

In addition to reductions in PTSD and alcohol use-related outcomes, participants experienced meaningful reductions in depressive symptoms during treatment, with average PHQ-9 scores decreasing from the moderately severe to the mild range of depression. These findings align with prior research showing that CPT reduces depressive symptoms, suicidal ideation, and hopelessness (Bryan et al., 2016; Resick et al., 2002). Given the high comorbidity between PTSD, AUD, and depression, the observed improvements across all three domains underscore the therapeutic potential of integrated CPT+RP treatment. Future studies with larger samples will help clarify whether these improvements in mood represent a direct treatment effect or are mediated by reductions in PTSD, alcohol use severity, or craving. The CPT+RP intervention was also well-tolerated and satisfaction ratings on the CSQ were high. This suggests that the integrated treatment was not only acceptable but also perceived by participants as valuable and relevant to their needs. This is noteworthy given the severity often observed among patients with co-occurring PTSD/AUD that is frequently linked to poor treatment engagement and high dropout rates.

Several limitations of the study should be noted. As a case series, the sample size was small and lacked a control group, precluding causal conclusions or any definitive statements about treatment efficacy. In addition, participants in the current study were a relatively homogenous group in terms of demographic characteristics and the findings may not generalize to more heterogeneous groups. Finally, the follow-up period was short and prevents conclusions about the long-term durability of treatment effects.

Nonetheless, this study provides encouraging early support for the CPT+RP intervention and suggests that it may be an acceptable and effective treatment for individuals with co-occurring PTSD and AUD. Strengths of the study include national participant recruitment, which enhances generalizability somewhat, use of telehealth, repeated standardized measures, both clinician-administered and self-report assessments, and use of a manualized treatment protocol. Future research might test the comparative effectiveness of treatment formats, including in-person, telehealth, or app-based delivery, on outcomes. The results are consistent with prior trials of trauma-focused integrated treatments (e.g., COPE) and suggest that CPT+RP may provide similar benefits, with the added advantage of offering a trauma-focused, non-exposure-based treatment approach. This has the potential to expand the menu of evidence-based, trauma-focused treatment options available to patients and providers, improve access to effective care, and ultimately enhance long-term recovery outcomes for this complex dual diagnosis. The ongoing randomized clinical trial (Vujanovic & Back, 2024) will provide a more rigorous evaluation of treatment efficacy and mechanisms of change. In addition, ecological momentary assessment (EMA) will capture dynamic interactions between PTSD symptoms, alcohol use, and craving in real time, enhancing our understanding of how these conditions influence one another during treatment.

## 5. Conclusions

Although preliminary, the findings from this case study demonstrate substantial promise for the feasibility, acceptability, and preliminary efficacy of a new integrated, trauma-focused CPT+RP intervention for co-occurring PTSD and AUD. Meaningful reductions were observed during treatment in PTSD symptoms, the frequency and amount of alcohol consumed, craving for alcohol, and depressive symptoms. The ongoing larger multisite RCT will determine if this study’s preliminary findings are replicated and how the integrated CPT+RP treatment compares to RP alone. Until then, these initial clinical cases show strong promise for the new trauma-focused, integrated therapy combining CPT and RP into a manualized protocol.

## Figures and Tables

**Table 1:** CPT+RP Treatment Session Content.

Session	Topic	CPT Focus	RP Focus
1	Introduction to Treatment	Overview of CPT/RP treatment, psychoeducation on PTSD/AUD comorbidity, goal setting, assign initial Impact Statement	
2	Examining the Impact of Trauma and Identifying Triggers for Alcohol Use	Review Impact Statement; identify trauma-related cognitive Stuck Points; examine connections among events, thoughts, and emotions	Identify alcohol and trauma-related triggers for alcohol craving and use
3	Working with Trauma-Relevant Thoughts/Feelings and Cravings	Review ABC ^a^ worksheets; challenge assimilated and overaccommodated cognitions about trauma	Review and normalize cravings and urges to drink; introduce distress tolerance skills to manage cravings and urges; identify alcohol-related cognitions with ABC worksheets
4	Examining the Index Event and High-Risk Thoughts About Using	Review Challenging Questions worksheet; differentiate among intention, responsibility, and the unforeseeable	Identify high-risk thoughts for alcohol use (e.g., Escape, Relaxation, Crisis, Control, Intimacy, Esteem)
5	Processing the Traumatic Event and Challenging Thoughts About Using	Use Challenging Questions worksheet; introduce Patterns of Problematic Thinking worksheet	Challenge and change high-risk thoughts about using alcohol; challenge patterns of thinking that increase risk of alcohol use
6	Learning to Self-Challenge and Tolerate Distress in Recovery	Review patterns of problematic trauma-related thinking and challenge negative thoughts	Review distress tolerance skills to manage cravings and thoughts about using
7	Learning to Self-Challenge and Cope with High-Risk Situations	Review Challenging Beliefs worksheet; review five trauma recovery themes; introduce Safety Theme	Identify and plan for high-risk situations and emergencies, distinguish lapse from relapse
8	Processing Safety and Building Refusal Skills	Review Challenging Beliefs worksheet on Safety; introduce Trust Theme	Review effective drink refusal skills; role play drink refusal scenarios
9	Processing Trust and Working with Negative Emotions	Review Challenging Beliefs worksheet on Trust; introduce Power/Control Theme	Identify negative emotions related to alcohol use; teach skills to work with negative emotions
10	Processing Power/Control and Urge Surfing	Review Challenging Beliefs worksheet on Power/Control; introduce Esteem Theme	Introduce Urge Surfing to tolerate and manage cravings to drink
11	Processing Esteem and Increasing Pleasant Activities and Support	Review Challenging Beliefs worksheet on Esteem; introduce Intimacy Theme; assign new Impact Statement	Increasing pleasant activities and cultivating support in recovery
12	Processing Intimacy, Final Impact Statement, Next Steps, and Termination	Review Challenging Beliefs worksheet on Intimacy; review original and new Impact Statements; discuss progress and potential next steps related to PTSD	Review progress; identify and plan for obstacles to recovery; discuss potential next steps related to AUD

Note. Each session includes: (a) Review patient’s alcohol use, cravings, and PTSD symptoms since last session; (b) Discuss any instances of alcohol use and conduct functional analysis to identify antecedents and consequences of use; (c) Connect trauma symptoms with alcohol craving and use; (d) Conduct Socratic questioning to address trauma- and alcohol use-related cognitions; and (e) Provide handouts/worksheets for assignments. ^a^ Activating Event (A), Belief/Stuck Point (B), Consequence (C).

**Table 2:** Means and Standard Deviations in PTSD Severity, Alcohol Use, and Depressive Symptoms.

Outcome	Assessment Timepoint			
	Baseline	Mid-Treatment	End-of-Treatment	3-Month Follow-Up
	*M* (*SD*)	*M* (*SD*)	*M* (*SD*)	*M* (*SD*)
CAPS-5	33.33 (4.50)	-	19.33 (14.50)	17.00 (5.02)
PCL-5	46.67 (10.27)	34.67 (22.06)	22.50 (16.93)	19.33 (11.41)
PDD	50.56 (23.54)	4.76 (11.66)	11.91 (29.16)	25.83 (25.67)
PHDD	48.32 (25.12)	4.76 (11.66)	11.90 (29.16)	23.61 (26.23)
ADD	7.29 (1.72)	1.50 (3.67)	1.05 (2.58)	3.40 (1.85)
PACS	17.33 (6.15)	14.50 (6.44)	11.17 (9.60)	7.50 (5.45)
PHQ-9	15.17 (8.23)	16.67 (6.25)	10.17 (7.49)	7.50 (9.13)

Note. CAPS-5 = Clinician Administered PTSD Scale for DSM-5; PCL-5 = PTSD Checklist for DSM-5; PDD = percent days drinking; PHDD = percent heavy days drinking; PACS = Penn Alcohol Craving Scale; ADD = average drinks per drinking day; PHQ-9 = Patient Health Questionnaire-9.

## Data Availability

Data are available upon request due to privacy and confidentiality protection.
